# Frailty and Clinical Outcomes in Patients Treated With Hemodialysis: A Prospective Cohort Study

**DOI:** 10.1016/j.xkme.2023.100684

**Published:** 2023-06-01

**Authors:** Marcello Tonelli, Natasha Wiebe, John S. Gill, Aminu K. Bello, Brenda R. Hemmelgarn, Christopher T. Chan, Anita Lloyd, Ravi I. Thadhani, Stephanie Thompson

**Affiliations:** 1 Department of Medicine, University of Calgary, Canada; 2 Department of Medicine, University of Alberta, Canada; 3 Department of Medicine, University of British Columbia, Canada; 4 Department of Medicine, University of Toronto, Canada; 5 Department of Medicine, Harvard University, MA

**Keywords:** Kidney failure, hemodialysis, frailty, chronic kidney disease, physical activity

## Abstract

**Rationale and Objective:**

Frailty is common among people with kidney failure treated with hemodialysis (HD). The objective was to describe how frailty evolves over time in people treated by HD, how improvements in frailty and frailty markers are associate with clinical outcomes, and the characteristics that are associated with improvement in frailty.

**Study Design:**

Prospective cohort study.

**Setting and Participants:**

Adults initiating thrice weekly in-center HD in Canada.

**Exposure:**

We classified frailty using a 5-point score (3 or more indicates frailty) based on physical inactivity, slowness or weakness, poor endurance or exhaustion, and malnutrition. We categorized the frailty trajectory as never present, improving, deteriorating, and always present.

**Outcomes:**

All-cause death, hospitalizations, and placement into long-term care.

**Analytical Approach:**

We examined the association between time-varying frailty measures and these outcomes using Cox and negative binomial models, after adjustment for potential confounders.

**Results:**

985 participants were included and followed up for a median of 33 months; 507 (51%) died, 761 (77%) experienced ≥1 hospitalization and 115 (12%) entered long-term care. Overall, 760 (77%) reported frailty during follow-up. Three-quarters (78%) of those with frailty at baseline remained frail throughout the follow-up, 46% without baseline frailty became frail, and 23% with baseline frailty became nonfrail. Higher frailty scores were associated with an increased risk of mortality (fully adjusted HR, 1.58 per unit; 95% CI, 1.39-1.80) and an increased rate of hospitalization (RR, 1.16 per unit; 95% CI, 1.09-1.23). Compared with those who were frail throughout the follow-up, participants with frailty at baseline but improving during follow-up showed a lower mortality (HR, 0.59; 95% CI, 0.42-0.81), and a lower rate of hospitalization (RR, 0.70; 95% CI, 0.56-0.87).

**Limitations:**

There was missing data on frailty at baseline and during follow-up.

**Conclusions:**

Frailty was associated with a higher risk of poor outcomes compared with those without frailty, and participants whose status improved from frail to nonfrail showed better clinical outcomes than those who remained frail. These findings emphasize the importance of identifying and implementing effective treatments for frailty in patients receiving maintenance HD.

## Graphical abstract

**Figure:**
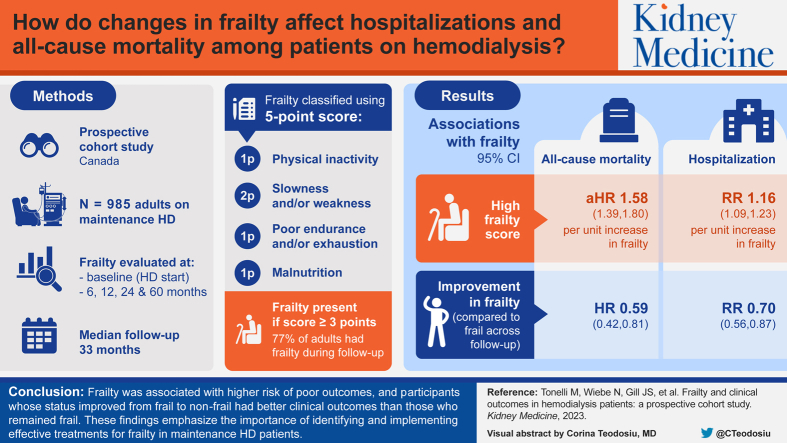

**Plain language summary**

Frailty is a syndrome of decreased physiological reserve and increased vulnerability to illness and death. We assessed 985 incident cases of patients receiving hemodialysis for frailty based on 4 characteristics at baseline and over up to 5-years of follow-up. The prevalence of frailty remained relatively stable over time, but the pattern of individual frailty characteristics did not. The proportion of participants with physical inactivity decreased, the proportion of participants with slowness, weakness or poor endurance or exhaustion remained stable, and the proportion with malnutrition increased. Frailty was associated with a higher risk of poor outcomes compared with those without frailty, and participants whose status improved from frail to nonfrail showed better clinical outcomes than those who remained frail. These findings may indicate the potential importance of early intervention to improve frailty after dialysis initiation.

Kidney failure treated with hemodialysis is associated with adverse clinical outcomes, high health care costs, and a poor quality of life.^1^ Frailty has been described as a syndrome of decreased physiological reserve, and increased vulnerability to illness and death.^2^ Frailty may be assessed in terms of physical capacity or alternatively in terms of cumulative deficits, which include assessment of comorbidity, disability, and symptoms.^3^ Regardless of the method used to assess it, frailty is common among people treated with hemodialysis,^4^ and frailty at the time of dialysis initiation is associated with excess risks of death and hospitalization.^5^ Other outcomes that have been associated with frailty in people treated with hemodialysis include a lower likelihood of receiving a kidney transplant, higher inpatient costs, and an increased risk of falls.^6^, ^7^, ^8^, ^9^

Less is known about how frailty evolves over time among people receiving maintenance hemodialysis, about how improvements in frailty markers are associated with clinical outcomes (compared to worsening in such markers), or about which characteristics are associated with improvement in frailty markers as opposed to deterioration.

We designed this study to investigate the evolution of frailty markers over time among incident cases of patients receiving hemodialysis, and how frailty markers (and changes in such markers over time) associate with clinical outcomes. The clinical outcomes we considered were death, hospitalization, and a new placement in a long-term care facility. The latter is a proxy for lost capacity to live independently, which is an outcome that is important to patients and their families.

## Methods

### Design

We did a secondary analysis of data from a prospective cohort study of patients receiving hemodialysis. Data (including frailty markers, as further mentioned) were collected from participant interviews, chart reviews, and clinical databases at baseline (start of hemodialysis),month 6, and year 1, 2, and 5. We ascertained demographics, medical and social history, weight, comorbidities, and the Kidney Disease Quality of Life (KDQOL) survey at baseline and updated them at each ensuing visit when and if the participants were receiving hemodialysis treatment. We tracked the modality transitions throughout the follow-up and included participants who returned to hemodialysis.

### Participants

Details of the Canadian Kidney Disease Cohort Study are presented elsewhere.^10^ We recruited eligible participants between March 2005, and November 2012, from participating in the hemodialysis programs based in 4 Canadian cities (Calgary, Edmonton, Ottawa, and Vancouver), and obtained written informed consents. The relevant research ethics boards approved the study (Pro00002385, REB15-1048, UBC-PHC REB H06-03483, and OHSN-REB 2009433-01H). We reported this study according to the STROBE guidelines.^11^

Adults (aged 18 years or older) initiating thrice weekly in-center hemodialysis were eligible for inclusion. We excluded participants who were not willing or unable to provide informed consent. In this analysis, we followed up participants until death, migration outside the study region, withdrawal of consent, switch to another modality, or study end (March 31, 2019), whichever was sooner.

Using a combination of interviews and chart reviews, study personnel collected data on demographic variables [age, sex, and ethnicity (White, indigenous, or otherwise)], body mass index, social variables [newcomer (immigrant or refugee), residence, employment], smoking status, primary cause of kidney failure (KF), and comorbidities (atrial fibrillation, myocardial infarction, heart failure, hypertension, peripheral vascular disease, dementia, cerebrovascular disease, diabetes mellitus, chronic obstructive pulmonary disease, cancers, liver diseases, psychiatric illness, and substance misuse). We gave paper forms to participants to self-report the KDQOL data.

### Frailty

We used a 5-point score for frailty published and validated by Johansen et al,^4^ where scores of 3, 4, or 5 indicate frailty, whereas a score of 0, 1, or 2 indicates nonfrail status (Item S1). The score has 4 components: physical inactivity, slowness and/or weakness, poor endurance and/or exhaustion, and malnutrition. There were 2 points allocated to slowness and/or weakness and 1 point for each of the other components.

The KDQOL is an instrument for assessing health-related quality of life in patients with KF, and includes 5 subscales: the physical component summary, mental component summary, burden of kidney disease, symptoms or problems of kidney disease, and the effects of kidney disease. Several self-reported measures of physical component summary are highly correlated with physical performance as measured on the original performance-based Fried score, and they are good predictors of adverse health outcomes.^4^^,^^12^ Consistent with Johansen et al, we defined 2 components of the frailty score as follows: (1) a score of <75/100 in the physical functioning domain of the KDQOL, which we used to define the presence of slowness and/or weakness, and (2) a score of <55/100 in the vitality domain of the KDQOL, which we used to define the presence of poor endurance and/or exhaustion. We assessed a third measure by asking participants how often they exercised or did physical activity during their leisure time. If they responded with “almost never or never,” they were considered physically inactive.

Malnutrition status was determined by linking with Alberta Health data. If there was a diagnosis of cachexia, weight loss or failure to thrive in any field in hospitalizations, provider claims, ambulatory care, or if the participant lost 10 kg of dry weight between visits, they were considered malnourished. Codes for cachexia were ICD-9 799.4 and ICD-10 R64. Codes for weight loss or failure to thrive were ICD-9 783.2, 783.3, 783.7 and ICD-10 R62.8, R63.3, R63.4. This state, once positive, was considered permanent unless the participant gained 10 kg of dry weight between visits. We attempted to assess these 4 components at each participant interview.

### Frailty Trajectories

We categorized the trajectory of the dichotomized frailty score for each participant with measures of 2 visits or more as never present, improving, deteriorating, and always present. With 3 or more visits, we regressed frailty status onto the month of visit, using mixed effects logistic regression with random intercepts for participants and random slopes for the month of visit. The calculated slopes from participants with only 2 visits were combined with the estimated slopes from participants with 3 visits or more. An improving trajectory meant that the slope was <-0.5 and a deteriorating trajectory meant that the slope was >0.5.

### Outcomes

Clinical outcomes were all-cause deaths, all-cause hospitalizations, and placement into long-term care. We ascertained death by chart review and ascertained the other outcomes by linking to the data from Alberta Health (the provincial health ministry). We excluded participants from outside Alberta (Ottawa and Vancouver) from all analyses of clinical outcomes except for death because these outcome data were not available. We determined that participants were residing in long-term care if they were discharged to a long-term care home after hospitalization, or if we identified 2 provider claims at least 30 days apart for services provided in a long-term care home; we deemed long-term care to have begun on the earlier of the date of discharge and the date of the first claim, respectively.

### Statistical Analyses

We did all analyses in Stata/MP 17.0 (www.stata.com). We reported descriptive statistics as counts and percentages, or medians and interquartile ranges, as appropriate. Tests for trend were calculated using logistic regression with random intercepts for participants when required. We regressed the clinical outcomes on time-varying frailty measures, using Cox and negative binomial models as appropriate. We further adjusted the models for baseline covariates: (1) age (18-40, 40-64, 65-79, and ≥80 years) and sex (female and male—no nonbinary participants were identified); (2) age, sex, newcomer status, ethnicity (White, Indigenous, and other), residence status (with family or friends, alone, assisted living/nursing home), employment status (employed full-time or full-time student or retiree, employed part-time or part-time student or homemaker or other, on disability, never employed, or currently unemployed), smoking status, body mass index (<18.5, 18.5-<26, 26-35, and ≥35 kg/m^2^), primary cause of KF (diabetic nephropathy, renal vascular disease, glomerulonephritis, polycystic kidney disease, other), and comorbidities—atrial fibrillation, myocardial infarction, chronic heart failure, hypertension, peripheral vascular disease, dementia, cerebrovascular accident, diabetes, chronic lung disease (including chronic obstructive pulmonary disease), cancer, chronic liver disease, psychiatric illness, and substance misuse; and 3) age, sex and the number of comorbidities (a maximum of 13). We coded missing values for baseline covariates with indicator variables. We did not fully adjust models where time to long-term placement was the outcome because there were too few events to appropriately fit the full complement of covariates. We also regressed the clinical outcomes on the frailty trajectories using the same modeling. We reported hazard ratios, rate ratios with 95% confidence intervals. In a sensitivity analysis, we imputed missing frailty components using chained equations^13^ (using logistic regression as the method) and missing trajectories using multinomial regression. The number of iterations was commensurate with the maximum fraction of missingness (60%).

## Results

### Participants

Participant flow is shown in Fig S1. Participant characteristics (Table 1) were similar to other Canadian incident cases of patients receiving hemodialysis.^14^ Seven percent of participants were from outside Alberta and so were excluded, leaving 985 participants with frailty measures who were included in this analysis (Fig S1). Over a median follow-up of 33 months (range 1-161 months), 507 (51%) participants died, 761 (77%) experienced ≥1 hospitalization (3,568 hospitalizations in total), and 115 (12%) were placed in long-term care [an additional 34 (3%) participants were in long-term care at baseline]. Sixty-one (6%) withdrew consent or migrated from Alberta during follow-up.

**Table 1:** Characteristics by Frailty Markers at First Visit With Measured Frailty

Characteristics	Frail	Not Frail	*P*
Participants	688 (69.8)	297 (30.2)	–
Age, y	64 (54-74)	61 (47-69)	<0.001
<40	60 (8.7)	42 (14.1)	–
40-64	297 (43.2)	135 (45.5)	–
65-79	261 (37.9)	95 (32.0)	**–**
≥80	70 (10.2)	25 (8.4)	**–**
Male	399 (58.0)	204 (68.7)	0.002
Ethnicity	–	–	0.005
White	562 (81.7)	221 (74.4)	–
Indigenous	36 (5.2)	13 (4.4)	–
Other	90 (13.1)	63 (21.2)	–
Newcomer	143 (21.3)	71 (24.5)	0.28
Residence	–	–	0.08
With family or friends	444 (69.8)	207 (74.7)	–
Alone	163 (25.6)	65 (23.5)	–
Assisted living/nursing home	29 (4.6)	5 (1.8)	–
Employment status	–	–	0.009
FT/FT student/retiree	402 (58.5)	188 (63.5)	–
PT/PT student/ homemaker/other	62 (9.0)	39 (13.2)	
On disability	175 (25.5)	49 (16.6)	
Never employed/ unemployed	48 (7.0)	20 (6.8)	
Smoker	124 (18.0)	43 (14.6)	0.18
BMI, kg/m^2^	27 (23-32)	25 (22-30)	<0.001
<18.5	24 (3.6)	17 (5.8)	
18.5-<26	274 (41.0)	153 (52.2)	
26-35	264 (39.5)	100 (34.1)	–
>35	106 (15.9)	23 (7.8)	–
Primary kidney failure	–	–	0.002
Diabetic nephropathy	297 (43.2)	100 (33.7)	**–**
Renal vascular disease	51 (7.4)	30 (10.1)	**–**
GN	85 (12.4)	53 (17.8)	**–**
PCKD	31 (4.5)	25 (8.4)	**–**
Other	224 (32.6)	89 (30.0)	**–**
Comorbidities	3 (2-4)	2 (2-3)	<0.001
Atrial fibrillation	121 (17.6)	29 (9.8)	0.002
AMI	180 (26.2)	45 (15.2)	<0.001
CHF	136 (19.8)	30 (10.1)	<0.001
Hypertension	605 (87.9)	261 (87.9)	0.98
PVD	76 (11.0)	25 (8.4)	0.21
Dementia	4 (0.6)	0 (0.0)	0.19
Cerebrovascular accident	97 (14.1)	36 (12.1)	0.41
Diabetes	415 (60.3)	159 (53.5)	0.048
Chronic lung disease	135 (19.6)	43 (14.5)	0.054
Cancer	93 (13.5)	29 (9.8)	0.10
Chronic liver disease	27 (3.9)	8 (2.7)	0.34
Psychiatric illness	117 (17.0)	29 (9.8)	0.003
Substance misuse	68 (10.9)	21 (7.6)	0.13

*Note:* n (%) and median (interquartile range) are reported.

Abbreviations: AMI, acute myocardial infarction; BMI, body mass index; CHF, chronic heart failure; FT, full-time; GN, glomerulonephritis; PCKD, polycystic kidney disease; PT, part-time; PVD, peripheral vascular disease.

### Frailty Markers

Because of incomplete or missing KDQOL forms, data on slowness and/or weakness and poor endurance and/or exhaustion were not always available (Table S1). The number of participant-visits with nonmeasured frailty components was greater in among non-White participants, and those with a number of comorbidities (diabetes, chronic lung disease, and psychiatric illness) or residing alone or in assisted living facilities.

Of the 985 participants, 835 (85%) at some point during the follow-up experienced slowness and/or weakness, 774 (79%) experienced poor endurance and/or exhaustion, 760 (77%) experienced physical inactivity, and 220 (22%) experienced malnutrition (Table S2). Overall, 775 (79%) were categorized as frail at some time during follow-up.

Compared with those with none of the frailty attributes, participants with one or more components of frailty at baseline were slightly older, more likely to be female, White, or residing in assisted living, and more likely to have diabetic nephropathy or a psychiatric illness, and more comorbidities generally.

**Figure 1:**
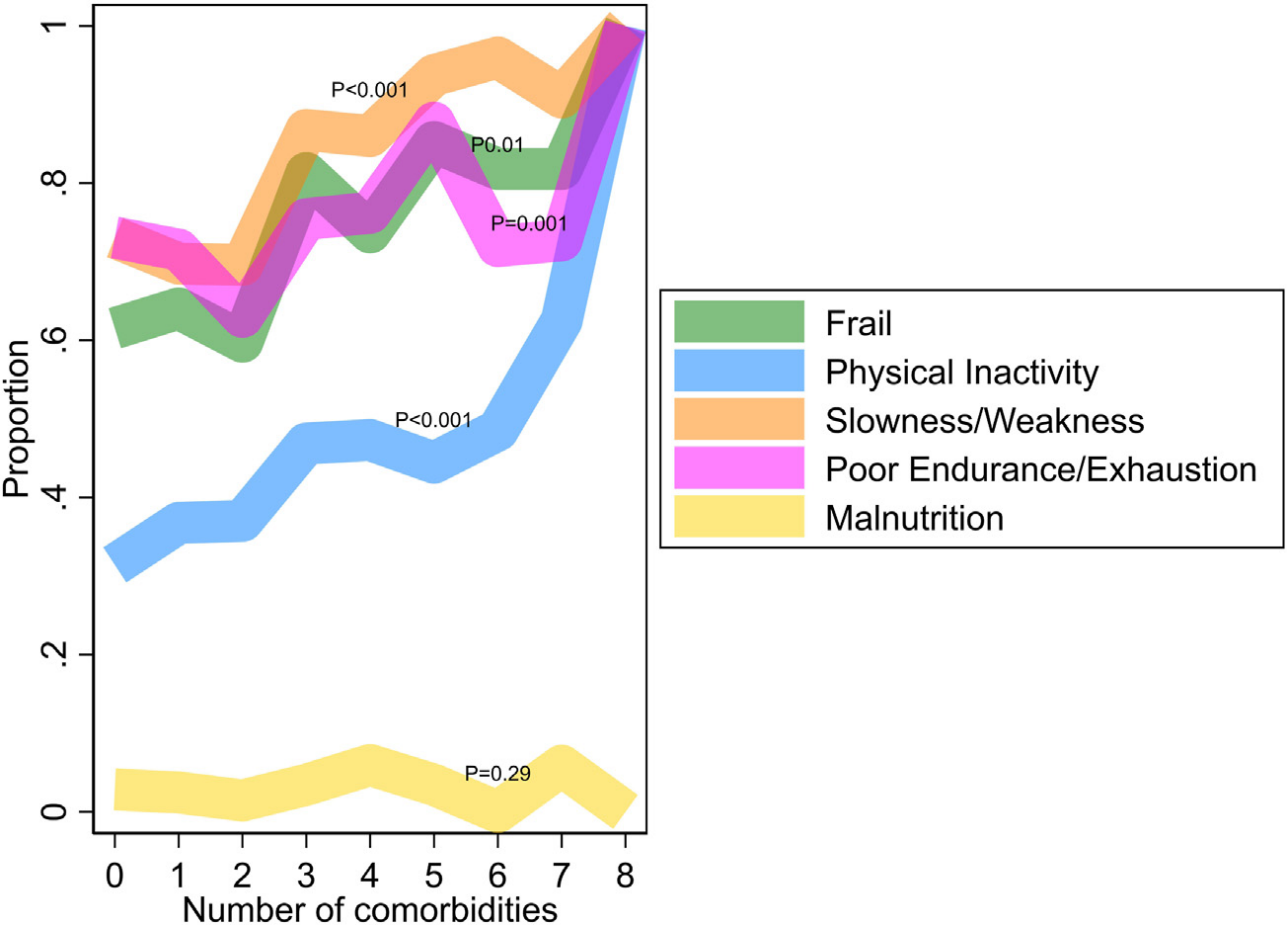
Proportion with frailty by the number of comorbidities at baseline level. The Figure shows the proportion of participants classified as frail based on a score of 3, 4, or 5 and the proportion with 4 individual components of the frailty score. One participant-visit with 9 comorbidities was excluded from this figure. Tests for trend were calculated using logistic regression.

The presence of slowness and/or weakness, poor endurance and/or exhaustion, and physical inactivity but not malnutrition were associated with a greater number of comorbidities as compared with those without these frailty characteristics, as was the overall presence of frailty (Fig 1). In addition, participants with physical inactivity were more likely to smoke and have chronic lung disease compared with those without physical inactivity.

### Temporal Changes in Frailty Markers

**Figure 2:**
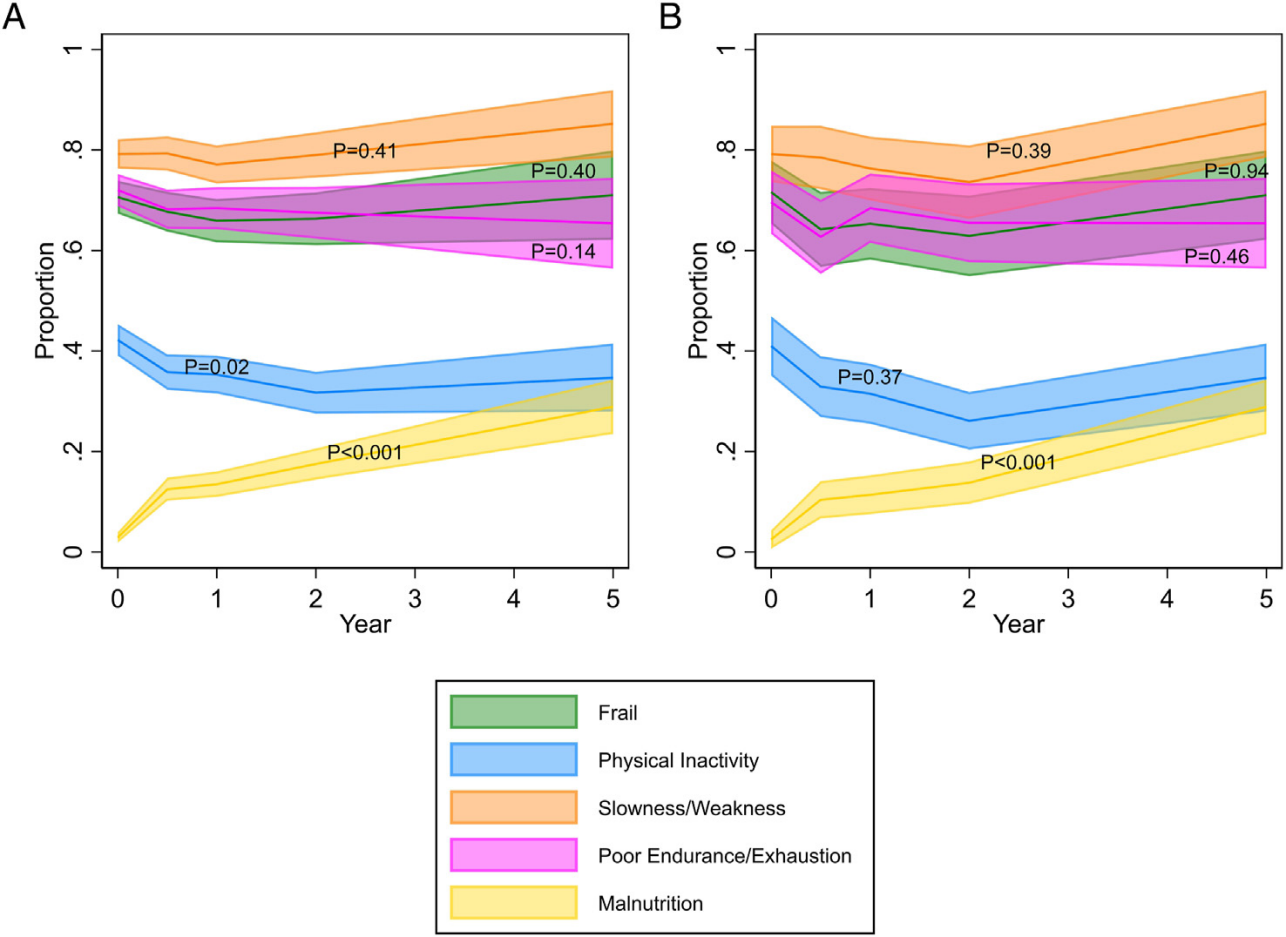
Domains across time. The Figure shows the proportion of participants classified as frail based on a score of 3, 4, or 5, and the proportion with 4 individual components of the frailty score. The colours show the width of 95% confidence limits. (A) The left panel is cross-sectional (with available data for any participant receiving hemodialysis, n=985). (B) The right panel is longitudinal (those who remain or return to hemodialysis in their fifth year, n=276). Tests for trend were calculated using logistic regression with a random intercept for participant.

The median frailty score remained constant over the course of follow-up, but there was some variability in the individual frailty components (Table 2). In particular, the proportion of participants with malnutrition increased over time (*P* < 0.001), whereas the proportion of participants with physical inactivity decreased (*P* = 0.02; Fig 2A). Results were similar in a complete case analysis that included only participants who were alive and contributed data after 5 years of follow-up (Fig 2B).

**Table 2:** Frailty Markers Over Time

Characteristic	Baseline	Month 6	Year 1	Year 2	Year 5	*P* for Linear Trend
Participants	985	805	706	578	270	–
Measurements (%)	744 (75.5)	536 (66.6)	466 (66.0)	312 (54.0)	100 (37.0)	–
Frailty score, median (IQR)	3 (2-4)	3 (2-4)	3 (2-4)	3 (2-4)	3 (2-4)	0.26
Frail (%)	525 (70.6)	363 (67.7)	307 (65.9)	207 (66.3)	71 (71.0)	0.40
Measurements (%)	950 (96.4)	713 (88.6)	620 (87.8)	482 (83.4)	190 (70.4)	–
Physical inactivity (%)	401 (42.2)	255 (35.8)	219 (35.3)	153 (31.7)	66 (34.7)	0.02
Measurements (%)	758 (77.0)	545 (67.7)	471 (66.7)	315 (54.5)	108 (40.0)	–
Slowness/weakness (%)	600 (79.2)	432 (79.3)	363 (77.1)	249 (79.0)	92 (85.2)	0.41
Measurements (%)	762 (77.4)	549 (68.2)	472 (66.9)	317 (54.8)	107 (39.6)	–
Poor endurance/exhaustion (%)	549 (72.0)	359 (65.4)	323 (68.4)	214 (67.5)	70 (65.4)	0.14
Malnutrition (%)	29 (2.9)	101 (12.5)	95 (13.5)	101 (17.5)	78 (28.9)	<0.001

*Note:* Each cell shows the number of participants (and percentage) with the characteristic measured, and the number of participants (and percentage) with that characteristic or the median (IQR) for the frailty score. The frailty score ranges from 0 to 5. High scores indicate more frailty. Malnutrition, by definition, had no missing measurements.

Abbreviations: IQR, interquartile range

### Associations Between Frailty Markers and Clinical Outcomes

Higher frailty scores were associated with an increased risk of mortality (fully adjusted HR, 1.58 per unit; 95% CI, 1.39-1.80) and an increased rate of hospitalization (RR, 1.16 per unit; 95% CI, 1.09-1.23) (Table 3). Fully adjusted models were not estimated for placement in long-term care, as explained in the Methods section.

**Table 3:** Clinical Outcomes by Frailty Markers

Models	Frailty Score, Per Unit Increase	Frail	Physical Inactivity	Slowness/Weakness	Poor Endurance/Exhaustion	Malnutrition
**All-cause mortality—Cox HR (95% CI)**						
Events	273	273	397	283	281	499
Participants/visits	985/2,154	985/2,154	985/2,949	985/2,193	985/2,203	985/3,324
Age-sex adjusted	1.57 (1.39-1.76)	1.87 (1.39-2.53)	1.75 (1.43-2.14)	3.50 (2.18-5.62)	1.37 (1.05-1.79)	1.20 (0.77-1.87)
Fully adjusted^a^	1.58 (1.39-1.80)	1.73 (1.26-2.38)	1.74 (1.41-2.14)	2.92 (1.79-4.76)	1.29 (0.97-1.71)	1.02 (0.64-1.61)
Adjustment for age, sex and number of comorbidities	1.52 (1.35-1.72)	1.73 (1.28-2.34)	1.69 (1.38-2.07)	3.07 (1.90-4.95)	1.31 (1.003-1.71)	1.24 (0.80-1.93)
Fully adjusted, with multiple imputation^a^	1.23 (1.12-1.36)	1.29 (1.02-1.64)	1.55 (1.27-1.89)	1.55 (1.12-2.14)	1.13 (0.90-1.43)	–
**All-cause hospitalizations—negative binomial RR (95% CI)**						
Events	2,045	2,045	3,031	2,087	2,098	3,526
Participants/visits	985/2,154	985/2,154	985/2,949	985/2,193	985/2,203	985/3,324
Age-sex adjusted	1.22 (1.15-1.29)	1.59 (1.36-1.86)	1.15 (1.03-1.30)	1.84 (1.49-2.27)	1.36 (1.16-1.58)	1.14 (0.67-1.95)
Fully adjusted^a^	1.16 (1.09-1.23)	1.40 (1.21-1.63)	1.10 (0.98-1.23)	1.58 (1.31-1.91)	1.18 (1.02-1.36)	1.10 (0.69-1.75)
Adjustment for age, sex and number of comorbidities	1.18 (1.11-1.26)	1.48 (1.27-1.73)	1.12 (1.0004-1.26)	1.67 (1.36-2.05)	1.28 (1.10-1.49)	1.12 (0.68-1.85)
Fully adjusted, with multiple imputation^a^	1.09 (1.03-1.15)	1.21 (1.06-1.39)	1.08 (0.97-1.21)	1.30 (1.09-1.54)	1.08 (0.94-1.23)	–
**Long-term care placement—Cox HR (95% CI)**						
Events	61	61	95	61	61	114
Participants/visits	943/2,033	943/2,033	949/2,775	943/2,069	943/2,078	951/3,098
Age-sex adjusted	1.63 (1.24-2.14)	3.34 (1.51-7.40)	1.32 (0.86-2.01)	2.97 (1.06-8.31)	2.26 (1.17-4.39)	1.12 (0.41-3.03)
Adjustment for age, sex and number of comorbidities	1.61 (1.22-2.12)	3.20 (1.44-7.12)	1.29 (0.84-1.96)	2.76 (0.98-7.78)	2.18 (1.12-4.24)	1.11 (0.41-3.01)
Adjustment for age, sex and number of comorbidities, with multiple imputation	1.22 (0.99-1.51)	1.79 (1.03-3.09)	1.22 (0.82-1.84)	1.51 (0.74-3.09)	1.48 (0.89-2.47)	–

*Note:* Frailty data were allowed to vary at baseline, month 6, and year1, 2, and 5 visits. No other covariates were allowed to vary across time.

Abbreviations: AMI, acute myocardial infarction; BMI, body mass index; CHF, chronic heart failure; CI, confidence interval; COPD, chronic obstructive pulmonary disease; FT, full-time; GN, glomerulonephritis; HR, hazard ratio; MI, multiple imputation; NA, not available; PCKD, polycystic kidney disease; PT, part-time; PVD, peripheral vascular disease; RR, rate ratio.

a The fully adjusted model was adjusted for baseline age (<40, 40-64, 65-79, ≥80 years), sex, newcomer status, ethnicity (White, indigenous, other), residence status (with family or friends, alone, assisted living/nursing home), employment status (employed FT or FT student or retiree, employed PT or PT student or homemaker or other, on disability, never employed or currently unemployed), smoking status, BMI (<18.5, 18.5-<26, 26-35, ≥35 kg/m^2^), primary cause of end-stage kidney disease (diabetic nephropathy, renal vascular disease, GN, PCKD, other), atrial fibrillation, AMI, CHF, hypertension, PVD, dementia, cerebrovascular accident, diabetes, chronic lung disease (including COPD), cancer, chronic liver disease, psychiatric illness, and substance misuse.

Adjustment for the number of comorbidities rather than the individual comorbidities showed little effect on the higher risk of mortality associated with higher frailty scores (HR, 1.52 per unit; 95% CI, 1.35-1.72) or rate of hospitalizations RR, 1.18 per unit; 95% CI, 1.11-1.26), and reported an association between frailty and the risk of placement in long-term care (HR, 1.61 per unit; 95% CI, 1.22-2.12). The age-sex-adjusted results were similar to those in more extensively adjusted models, suggesting that the association between frailty and clinical outcomes was largely independent from measured comorbidity.

In analyses that imputed the missing frailty measures, frailty remained significantly associated with mortality and hospitalizations: (mortality HR, 1.23 per unit; 95% CI, 1.12-1.36; hospitalizations RR, 1.09 per unit; 95% CI, 1.03-1.15) but not placement into long-term care (HR, 1.22 per unit; 95% CI, 0.99-1.51).

When considered individually, slowness and/or weakness and physical inactivity were associated with the risk of mortality (HR, 2.92; 95% CI, 1.79-4.76, and HR, 1.74; 95% CI, 1.41-2.14, respectively) and slowness and/or weakness was associated with rate of hospitalizations (RR, 1.58; 95% CI, 1.31-1.91). Poor endurance and/or exhaustion was associated with risk of hospitalizations (RR, 1.18; 95% CI, 1.02-1.36).

### Associations Between Temporal Changes in Frailty Markers and Clinical Outcomes

Three-quarters (78%) of those with frailty at baseline remained frail throughout follow-up, 46% without baseline frailty became frail, and 23% with baseline frailty became nonfrail.

Compared with those who were frail throughout the follow-up, participants who were frail at baseline but improved over follow-up showed a lower mortality rate (HR, 0.57; 95% CI, 0.40-0.80), and showed a lower rate of hospitalization (RR, 0.70; 95% CI, 0.56-0.87) (Table S3).

Compared with those who remained free of frailty throughout the follow-up (never frail), participants who were not initially frail but became frail over follow-up showed a significantly higher rate of hospitalization (RR, 1.44; 95% CI, 1.13-1.82), but no significant difference in the rate of mortality or placement into long-term care. Results were again similar in analyses that imputed the missing frailty measures.

## Discussion

This analysis of frailty and its clinical correlates among 985 incident cases of patients receiving hemodialysis reported 4 main findings. First, participants with frailty were more likely to be older, female, White, or to have more comorbidity at baseline, including a history of psychiatric illness. Second, although the overall prevalence of frailty remained relatively stable over time, the pattern of the individual frailty components did not. The proportion of participants with physical inactivity slightly decreased over time, whereas the proportion of participants with slowness, weakness or poor endurance or exhaustion remained stable and the proportion with malnutrition increased. These temporal changes in prevalence could have been partially because of survivorship bias. However, the finding that 46% of those who were not initially frail and 23% of those who were frail at baseline changed their status during follow-up suggests that true changes in frailty also contributed to the results. Third, higher levels of frailty were independently associated with a higher likelihood of adverse clinical outcomes (death, hospitalization, and placement in long-term care). Fourth, participants whose status improved from frail to nonfrail during the follow-up showed better clinical outcomes than those who remained frail throughout the follow-up, and those whose status deteriorated from nonfrail to frail reported higher mortality than those who did not. These findings may indicate the potential importance of early intervention to improve frailty soon after dialysis initiation, although this suggestion is speculative.

Johansen et al^4^ studied a nationally representative group of 2275 incident cases of US patients receiving hemodialysis and found that frailty (as defined by Fried et al^15^) was present in 67.7% within ∼60 days of dialysis initiation, similar to 72.2% at baseline in this study. Johansen et al found that frailty was independently associated with 1-year mortality. Bao et al,^5^ studied a second cohort of 1576 incident cases of US patients receiving hemodialysis, and found that 73% were frail at dialysis initiation; frailty was independently associated with the risks of mortality (median follow-up of 2.9 years) and hospitalization (median follow-up of 1.2 years). Multiple other studies have confirmed an association between frailty and mortality in patients receiving hemodialysis.^16^^,^^17^ Our findings extend results from previous work by documenting longitudinal trajectories of frailty over as long as 5 years, evaluating the independent contribution of frailty trajectories in addition to the baseline presence or absence of frailty, and studying the association between frailty and placement in a long-term care facility, an outcome that is important to patients and families.

Although some have suggested that dialysis patients should be routinely evaluated for frailty,^18^ the utility of this approach is uncertain given the lack of proven interventions. Candidate therapies, such as exercise training,^19^ androgen supplementation,^20^ carnitine administration,^21^ and perhaps other forms of nutritional support.^22^ The high prevalence of frailty, the high associated costs,^23^ and the excess risk of associated adverse outcomes indicate the need for clinical trials to identify effective treatments.

Our study has several important strengths, such as its relatively large size, prospective multi-center design, validated measure of frailty, and standardized assessment of clinical characteristics and outcomes. However, our study also has limitations that should be considered when interpreting its findings. First, some participants showed missing data on frailty status, generally because they declined to complete the relevant study questionnaires, such as the KDQOL. Although results were consistent in a battery of sensitivity analyses, the findings remain potentially vulnerable to response bias. Missing data during follow-up were more common among those with frailty at baseline but also among those who received a kidney transplant or transitioned to peritoneal dialysis, and therefore the direction of any such bias is unpredictable. Second, like all longitudinal studies of patients receiving hemodialysis, our results could have been influenced by survivorship effects. However, because we observed changes in frailty status among survivors, it is unlikely that the temporal changes in prevalence were solely because of differential frailty status among those with early deaths when compared with those who were followed up for a longer period. Third, despite our best efforts to adjust for important confounders, the potential for residual confounding remains. Fourth, although we used accepted measures of frailty to classify the exposure, the individual markers are non-specific and arguably could reflect the burden of comorbidity or overall health rather than the more nuanced construct of frailty per se. In support of this hypothesis, frailty in incident cases of patients receiving hemodialysis has been associated with a wide range of adverse outcomes, including prolonged postdialysis recovery time,^24^ vascular access thrombosis,^25^ and perhaps more severe metabolic bone disease.^26^ Fifth, previous work suggests that the choice of frailty measure will influence the apparent prevalence of frailty, which should be considered when interpreting our results.^27^ For example, using an objective measure of frailty as opposed to self-report tends to lead to a lower prevalence of frailty than reported herein.^28^ Sixth, our definition of physical inactivity was based on self-reported activity during leisure time only, and a more comprehensive assessment (ie, all self-reported activity) might have been preferable. Finally, our study population was drawn from Canadian patients receiving hemodialysis and may not apply to other settings.

In conclusion, our findings suggest that frailty is common at the time of hemodialysis initiation, and the overall prevalence of frailty remains relatively stable in longitudinal analyses, although the prevalence of the individual frailty components does not, possibly because of survivorship bias as well as true changes in clinical status. Higher levels of frailty at baseline and during follow-up are independently associated with a higher likelihood of death, hospitalization, and placement in long-term care, and improvements in frailty status are associated with better clinical outcomes than remaining frail. These findings emphasize the importance of identifying and implementing effective treatments for frailty in patients receiving maintenance hemodialysis.

## Supplementary Material

Supplementary File (PDF) Figure S1; Item S1; Table S1-S3.
