# Supplementary material for: Frailty and Clinical Outcomes in Patients Treated With Hemodialysis: A Prospective Cohort Study
Source: Kidney Med. 2023 Jun 1;5(8):100684. doi: 10.1016/j.xkme.2023.100684 (PMC10368915; doi:10.1016/j.xkme.2023.100684)
Supplement: Supplementary File (PDF) — Figure S1; Item S1; Table S1-S3. [file mmc1.pdf]

Figure S1. Participant flow diagram

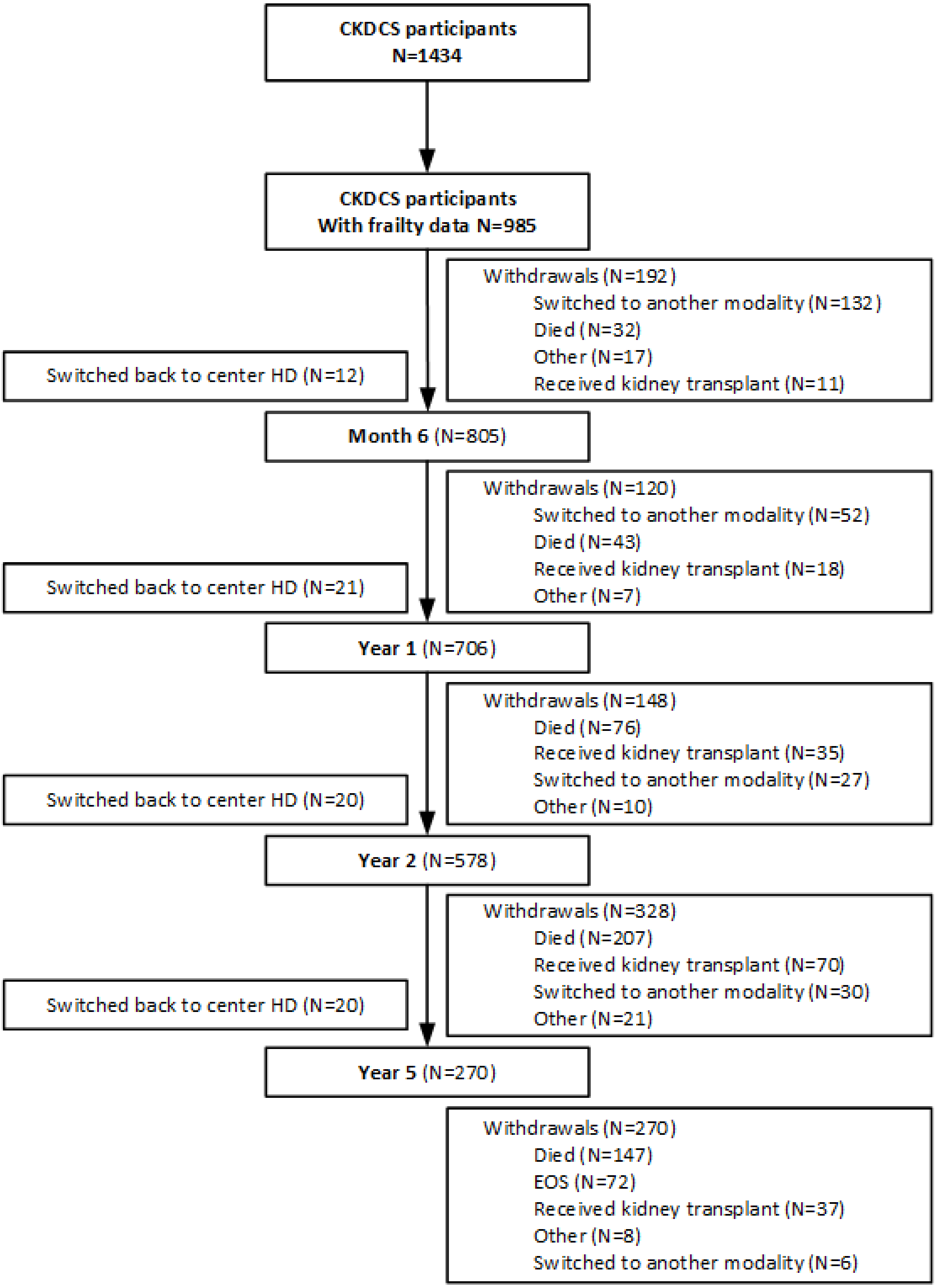

CKDCS Canadian Kidney Disease Cohort Study, EOS end of study, HD hemodialysis

Participants could withdraw from the cohort due to a switch in modality or a kidney transplant and then return to the cohort if they switched back to hemodialysis.

Item S1. Frailty score

Frailty	Score (5)
Physical Inactivity<br>CKDCS survey	1
Slowness and/or Weakness<br>KDQOL physical functioning <75	2
Poor Endurance and/or Exhaustion<br>KDQOL vitality <55	1
Malnutrition<br>ICD-9 to ICD-10-CA codes	1

Table S1. Characteristics by frailty markers and missingness across all participant-visits (N=1434 participants, 5566 visits)

Characteristics	Physical inactivity Measured	Not measured	Slowness/ weakness Measured	Not measured	Poor endurance/ exhaustion Measured	Not measured
Measurements	4,344 (78.1)	1,222 (22.0)	2,514 (45.2)	3,052 (54.8)	2,541 (45.7)	3,025 (54.3)
Age, years	64 [53,74]	64 [54,74]	64 [53,74]	64 [53,74]	64 [53,74]	64 [53,74]
<40	409 (9.4)	131 (10.7)	229 (9.1)	311 (10.2)	231 (9.1)	309 (10.2)
40-64	1,803 (41.5)	481 (39.4)	1,051 (41.8)	1,233 (40.4)	1,061 (41.8)	1,223 (40.4)
65-79	1,557 (35.8)	462 (37.8)	932 (37.1)	1,087 (35.6)	944 (37.2)	1,075 (35.5)
≥80	575 (13.2)	148 (12.1)	302 (12.0)	421 (13.8)	305 (12.0)	418 (13.8)
Male	2,661 (61.3)	733 (60.0)	1,528 (60.8)	1,866 (61.1)	1,545 (60.8)	1,849 (61.1)
Ethnicity						
White	3,242 (74.6)	856 (70.0)	1,977 (78.6)	2,121 (69.5)	2,004 (78.9)	2,094 (69.2)
Indigenous	301 (6.9)	128 (10.5)	125 (5.0)	304 (10.0)	124 (4.9)	305 (10.1)
Other	801 (18.4)	238 (19.5)	412 (16.4)	627 (20.5)	413 (16.3)	626 (20.7)
Newcomer	1,079 (25.3)	230 (21.0)	577 (23.4)	732 (25.3)	582 (23.4)	727 (25.3)
Residence						
With family or friends	2,969 (71.4)	122 (68.2)	1,655 (70.6)	1,436 (72.0)	1,677 (70.8)	1,414 (71.8)
Alone	904 (21.7)	29 (16.2)	551 (23.5)	382 (19.2)	555 (23.4)	378 (19.2)
Assisted living/nursing home	286 (6.9)	28 (15.6)	138 (5.9)	176 (8.8)	138 (5.8)	176 (8.9)
Employment status						
FT/FT student/retiree	2,596 (60.3)	86 (60.1)	1,511 (61.9)	1,171 (58.4)	1,524 (61.8)	1,158 (58.5)
PT/PT student/ homemaker/other	348 (8.1)	15 (10.5)	203 (8.3)	160 (8.0)	205 (8.3)	158 (8.0)
On disability	1,109 (25.8)	31 (21.7)	581 (23.8)	559 (27.9)	591 (24.0)	549 (27.7)
Never employed/ unemployed	249 (5.8)	11 (7.7)	146 (6.0)	114 (5.7)	146 (5.9)	114 (5.8)
Smoker	770 (17.8)	189 (17.9)	449 (17.9)	510 (17.7)	450 (17.8)	509 (17.8)
BMI, kg/m^2	26 [23,31]	26 [22,31]	27 [23,31]	26 [22,31]	27 [23,31]	26 [22,31]
<18.5	205 (4.8)	75 (6.4)	103 (4.2)	177 (5.9)	103 (4.1)	177 (6.0)
18.5-25	1,826 (42.8)	515 (43.6)	1,043 (42.2)	1,298 (43.5)	1,049 (42.0)	1,292 (43.7)
26-35	1,687 (39.5)	443 (37.5)	990 (40.1)	1,140 (38.2)	1,007 (40.3)	1,123 (38.0)
>35	552 (12.9)	148 (12.5)	334 (13.5)	366 (12.3)	338 (13.5)	362 (12.3)
Primary kidney failure						
Diabetic nephropathy	1,867 (43.0)	532 (43.5)	1,022 (40.7)	1,377 (45.1)	1,038 (40.9)	1,361 (45.0)
Renal vascular disease	375 (8.6)	79 (6.5)	215 (8.6)	239 (7.8)	219 (8.6)	235 (7.8)
GN	587 (13.5)	194 (15.9)	346 (13.8)	435 (14.3)	347 (13.7)	434 (14.3)
PCKD	207 (4.8)	60 (4.9)	146 (5.8)	121 (4.0)	148 (5.8)	119 (3.9)
Other	1,308 (30.1)	357 (29.2)	785 (31.2)	880 (28.8)	789 (31.1)	876 (29.0)
Comorbidities	3 [2,4]	3 [2,4]	3 [2,4]	3 [2,4]	3 [2,4]	3 [2,4]
Atrial fibrillation	879 (20.3)	246 (20.2)	427 (17.0)	524 (17.2)	436 (17.2)	515 (17.0)
AMI	276 (21.8)	33 (19.5)	601 (23.9)	668 (21.9)	605 (23.8)	664 (22.0)
CHF	942 (21.7)	296 (24.3)	462 (18.4)	641 (21.0)	470 (18.5)	633 (20.9)
Hypertension	1,108 (87.8)	144 (85.7)	2,216 (88.1)	2,752 (90.2)	2,239 (88.1)	2,729 (90.2)
PVD	120 (9.5)	22 (13.0)	266 (10.6)	356 (11.7)	272 (10.7)	350 (11.6)
Dementia	156 (3.6)	90 (7.4)	16 (0.6)	60 (2.0)	16 (0.6)	60 (2.0)
Cerebrovascular accident	193 (15.3)	19 (11.2)	326 (13.0)	406 (13.3)	333 (13.1)	399 (13.2)
Diabetes	2,676 (61.6)	751 (61.5)	1,491 (59.3)	1,936 (63.4)	1,511 (59.5)	1,916 (63.3)
Chronic lung disease	926 (21.3)	288 (23.6)	509 (20.2)	705 (23.1)	519 (20.4)	695 (23.0)
Cancer	503 (11.6)	174 (14.3)	282 (11.2)	395 (12.9)	285 (11.2)	392 (13.0)

Characteristics	Physical inactivity Measured	Not measured	Slowness/ weakness Measured	Not measured	Poor endurance/ exhaustion Measured	Not measured
Chronic liver disease	131 (3.0)	45 (3.7)	76 (3.0)	100 (3.3)	77 (3.0)	99 (3.3)
Psychiatric illness	723 (16.7)	232 (19.2)	391 (15.6)	565 (18.5)	400 (15.7)	556 (18.4)
Substance misuse	214 (6.4)	50 (6.7)	178 (7.7)	194 (6.7)	179 (7.7)	193 (6.8)

AMI acute myocardial infarction, BMI body mass index, CHF chronic heart failure, FT full-time, GN glomerulonephritis, NA not available, PCKD polycystic kidney disease, PT part-time, PVD peripheral vascular disease

N (%) and median [interquartile range] are reported.

Table S2. Characteristics by frailty components

Characteristics	Baseline All	At first visit with Frailty	Physical inactivity	Slowness/ weakness	Poor endurance/ exhaustion	Malnutriti[illegible]
Participants	985	760	583	835	774	202
Age, years	63 [51,73]	64 [54,74]	64 [53,74]	64 [54,74]	63 [52,74]	67 [59,76]
<40	103 (10.5)	64 (8.4)	53 (9.1)	70 (8.4)	78 (10.1)	9 (4.5)
40-64	445 (45.2)	324 (42.6)	248 (42.5)	349 (41.8)	340 (43.9)	75 (37.1)
65-79	347 (35.2)	294 (38.7)	218 (37.4)	326 (39.0)	278 (35.9)	87 (43.1)
≥80	90 (9.1)	78 (10.3)	64 (11.0)	90 (10.8)	78 (10.1)	31 (15.3)
Male	604 (61.3)	446 (58.7)	334 (57.3)	496 (59.4)	460 (59.4)	125 (61.9)
Ethnicity						
White	781 (79.3)	622 (81.8)	470 (80.6)	670 (80.2)	632 (81.7)	168 (83.2)
Indigenous	49 (5.0)	37 (4.9)	28 (4.8)	44 (5.3)	37 (4.8)	7 (3.5)
Other	155 (15.7)	101 (13.3)	85 (14.6)	121 (14.5)	105 (13.6)	27 (13.4)
Newcomer	211 (22.0)	158 (21.4)	128 (22.5)	181 (22.2)	153 (20.3)	48 (23.9)
Residence						
With family or friends	649 (72.8)	488 (69.4)	384 (70.5)	543 (70.5)	493 (69.3)	125 (65.8)
Alone	218 (24.5)	185 (26.3)	136 (25.0)	194 (25.2)	192 (27.0)	43 (22.6)
Assisted living/nursing home	24 (2.7)	30 (4.3)	25 (4.6)	33 (4.3)	26 (3.7)	22 (11.6)
Employment status						
FT/FT student/retiree	573 (59.5)	454 (59.8)	345 (59.2)	498 (60.1)	452 (58.8)	126 (63.3)
PT/PT student/ homemaker/other	101 (10.5)	67 (8.8)	53 (9.1)	75 (9.0)	73 (9.5)	11 (5.5)
On disability	226 (23.5)	185 (24.4)	147 (25.2)	195 (23.5)	189 (24.6)	56 (28.1)
Never employed/ unemployed	63 (6.5)	53 (7.0)	38 (6.5)	61 (7.4)	55 (7.2)	6 (3.0)
Smoker	166 (16.9)	135 (17.8)	114 (19.6)	139 (16.7)	134 (17.3)	32 (15.8)
BMI, kg/m^2	26 [23,31]	27 [23,32]	27 [23,32]	26 [23,31]	27 [23,32]	26 [21,30]
<18.5	42 (4.4)	28 (3.8)	29 (5.1)	33 (4.0)	31 (4.1)	16 (8.0)
18.5-<26	427 (44.4)	309 (41.8)	221 (38.8)	352 (43.2)	315 (41.7)	84 (42.2)
26-35	360 (37.4)	287 (38.8)	223 (39.2)	311 (38.2)	292 (38.7)	76 (38.2)
>35	133 (13.8)	116 (15.7)	96 (16.9)	119 (14.6)	117 (15.5)	23 (11.6)
Primary kidney failure						
Diabetic nephropathy	397 (40.3)	328 (43.2)	260 (44.6)	361 (43.2)	316 (40.8)	88 (43.6)
Renal vascular disease	81 (8.2)	56 (7.4)	44 (7.5)	67 (8.0)	55 (7.1)	18 (8.9)
GN	138 (14.0)	96 (12.6)	79 (13.6)	100 (12.0)	106 (13.7)	24 (11.9)
PCKD	56 (5.7)	34 (4.5)	33 (5.7)	37 (4.4)	44 (5.7)	8 (4.0)
Other	313 (31.8)	246 (32.4)	167 (28.6)	270 (32.3)	253 (32.7)	64 (31.7)
Comorbidities	3[2,4]	3[2,4]	3 [2,4]	3[2,4]	3[2,4]	3 [2,4]
Atrial fibrillation	140 (14.2)	136 (17.9)	91 (15.6)	145 (17.4)	130 (16.8)	62 (30.7)
AMI	225 (22.8)	195 (25.7)	144 (24.7)	209 (25.0)	195 (25.2)	52 (25.7)
CHF	165 (16.8)	147 (19.3)	104 (17.8)	159 (19.0)	139 (18.0)	53 (26.2)
Hypertension	866 (87.9)	669 (88.0)	520 (89.2)	735 (88.0)	681 (88.0)	176 (87.1)
PVD	101 (10.3)	87 (11.4)	66 (11.3)	96 (11.5)	82 (10.6)	20 (9.9)
Dementia	5 (0.5)	6 (0.8)	4 (0.7)	5 (0.6)	3 (0.4)	3 (1.5)
Cerebrovascular accident	133 (13.5)	108 (14.2)	91 (15.6)	124 (14.9)	104 (13.4)	25 (12.4)
Diabetes	572 (58.1)	457 (60.1)	353 (60.5)	506 (60.6)	454 (58.7)	129 (63.9)
Chronic lung disease	169 (17.2)	153 (20.1)	130 (22.3)	163 (19.5)	150 (19.4)	62 (30.7)
Cancer	121 (12.3)	101 (13.3)	80 (13.7)	107 (12.8)	98 (12.7)	35 (17.3)
Chronic liver disease	38 (3.9)	28 (3.7)	22 (3.8)	30 (3.6)	31 (4.0)	4 (2.0)
Psychiatric illness	126 (12.8)	124 (16.3)	109 (18.7)	131 (15.7)	125 (16.1)	33 (16.3)
Substance misuse	98 (10.9)	69 (10.0)	48 (9.1)	76 (10.0)	70 (9.9)	13 (6.9)

AMI acute myocardial infarction, BMI body mass index, CHF chronic heart failure, FT full-time, GN glomerulonephritis, PCKD polycystic kidney disease, PT part-time, PVD peripheral vascular disease
N (%) and median [interquartile range] are reported. The following characteristics were missing data at baseline: 26 (2.6%) newcomer, 94 (9.5%) residence, 22 (2.2%) employment, 4 (0.4%) smoking, 23 (2.3%) BMI, and 88 (8.9%) substance misuse. There were 985 participants included in the study; 760 were assessed as frail at some visit during follow-up including 835 participants assessed as slow and/or weak, 774 with exhaustion or poor endurance, 583 as physically inactive, and 202 as malnourished.

Table S3. Clinical outcomes by frailty trajectories

Models	Never frail	Improving	Deteriorating	Always frail
All-cause mortality – Cox HR (95% CI)				
Events (%)	41 (38.0)	46 (48.4)	51 (56.0)	208 (65.2)
Fully adjusted[1]	0.51 (0.35,0.73)	0.57 (0.40,0.80)	0.57 (0.42,0.79)	1.00 (referent)
Age-sex & no. of comorbidities	0.58 (0.42,0.82)	0.59 (0.42,0.81)	0.60 (0.44,0.82)	1.00 (referent)
MI fully adjusted[1]	0.61 (0.44,0.86)	0.70 (0.50,0.97)	0.72 (0.54,0.95)	1.00 (referent)
All-cause hospitalizations – Negative binomial RR (95% CI)				
Events (rate per year)	249 (19.8)	372 (22.8)	673 (30.5)	1297 (33.0)
Fully adjusted[1]	0.67 (0.55,0.82)	0.70 (0.56,0.87)	0.96 (0.80,1.17)	1.00 (referent)
Age-sex & no. of comorbidities	0.63 (0.50,0.79)	0.67 (0.53,0.84)	0.93 (0.76,1.12)	1.00 (referent)
MI fully adjusted[1]	0.77 (0.63,0.94)	0.78 (0.63,0.96)	0.98 (0.82,1.16)	1.00 (referent)
Long-term care placement - Cox HR (95% CI)				
Events (%)	5 (5.6)	10 (10.9)	17 (14.4)	47 (16.0)
Age-sex & no. of comorbidities	0.50 (0.22,1.12)	0.49 (0.24,1.01)	0.59 (0.31,1.11)	1.00 (referent)
MI age-sex & no. of comorbidities	0.59 (0.28,1.25)	0.54 (0.28,1.04)	0.71 (0.39,1.28)	1.00 (referent)

AMI acute myocardial infarction, BMI body mass index, CHF chronic heart failure, COPD chronic obstructive pulmonary disease, FT full-time, GN glomerulonephritis, HR hazard ratio, MI multiple imputation, NA not available, PCKD polycystic kidney disease, PT part-time, PVD peripheral vascular disease, RR rate ratio

Frailty trajectories were determined from at least 2 measures.

[1]The fully adjusted model was adjusted for: baseline age (<40, 40-64, 65-79, ≥80 years), sex, newcomer status, ethnicity (white, Indigenous, other), residence status (with family or friends, alone, assisted living/nursing home), employment status (employed FT or FT student or retiree, employed PT or PT student or homemaker or other, on disability, never employed or currently unemployed), smoking status, BMI (<18.5, 18.5-<26, 26-35, ≥35 kg/m^2), primary cause of end-stage renal disease (diabetic nephropathy, renal vascular disease, GN, PCKD, other), atrial fibrillation, AMI, CHF, hypertension, PVD, dementia, cerebrovascular accident, diabetes, chronic lung disease (including COPD), cancer, chronic liver disease, psychiatric illness, and substance misuse.
